# Toward Genomics-Based Breeding in C3 Cool-Season Perennial Grasses

**DOI:** 10.3389/fpls.2017.01317

**Published:** 2017-07-26

**Authors:** Shyamal K. Talukder, Malay C. Saha

**Affiliations:** Noble Research Institute, LLC, Ardmore OK, United States

**Keywords:** perennial grass, marker-assisted selection, genomic selection, next-generation sequencing, QTL mapping

## Abstract

Most important food and feed crops in the world belong to the C3 grass family. The future of food security is highly reliant on achieving genetic gains of those grasses. Conventional breeding methods have already reached a plateau for improving major crops. Genomics tools and resources have opened an avenue to explore genome-wide variability and make use of the variation for enhancing genetic gains in breeding programs. Major C3 annual cereal breeding programs are well equipped with genomic tools; however, genomic research of C3 cool-season perennial grasses is lagging behind. In this review, we discuss the currently available genomics tools and approaches useful for C3 cool-season perennial grass breeding. Along with a general review, we emphasize the discussion focusing on forage grasses that were considered orphan and have little or no genetic information available. Transcriptome sequencing and genotype-by-sequencing technology for genome-wide marker detection using next-generation sequencing (NGS) are very promising as genomics tools. Most C3 cool-season perennial grass members have no prior genetic information; thus NGS technology will enhance collinear study with other C3 model grasses like *Brachypodium* and rice. Transcriptomics data can be used for identification of functional genes and molecular markers, i.e., polymorphism markers and simple sequence repeats (SSRs). Genome-wide association study with NGS-based markers will facilitate marker identification for marker-assisted selection. With limited genetic information, genomic selection holds great promise to breeders for attaining maximum genetic gain of the cool-season C3 perennial grasses. Application of all these tools can ensure better genetic gains, reduce length of selection cycles, and facilitate cultivar development to meet the future demand for food and fodder.

## Introduction

There are three photosynthetic carbon fixation pathways in plants. In one pathway, plants fix atmospheric carbon dioxide (CO_2_) using Rubisco in Calvin cycle to produce a first stable product as three carbon compounds with no photosynthetic adaptation to reduce photorespiration. This group of plants is called C3 plants. In the second pathway, plants produce four carbon compounds as their first stable product and minimize photorespiration by separating the Calvin cycle from the light-dependent reaction using a physical barrier. This group of plants is called C4 plants. In the third pathway, plants use time to separate the light-dependent reaction instead of a physical barrier. This pathway was first discovered in the Crassulaceae family, thus it is called the Crassulacean Acid Metabolism (CAM) pathway. Plants which use this pathway are called CAM plants (Raines, 2011).

The Poaceae family, commonly known as the grass family, is an important and widely distributed plant group on Earth (Stevens, 2001). Several members of this family are the most economically important plants supporting food, feed, industry, and lawns. The majority of these grasses belong to the C3 plant group (Araus et al., 2002). Bread wheat (*Triticum aestivum*) and rice (*Oryza sativa*), two out of the three most important staple food crops in the world, along with barley (*Hordeum vulgare*), rye (*Secale cereale*), oat (*Avena sativa*), and millet (*Pennisetum glaucum*), belong to the C3 grasses. Almost all cool-season perennial grasses used as forages and turf, i.e., orchardgrass (*Dactylis glomerata*), fescue (*Festuca* spp.), Kentucky bluegrass (*Poa pratensis*), perennial ryegrass (*Lolium perenne*), bentgrass (*Agrostis* spp.), Phalaris (*Phalaris aquatica* L.), intermediate wheatgrass (*Thinopyrum intermedium*), and sheepgrass [*Leymus chinensis* (Trin.)], are also C3 grasses (Bonos et al., 2006). *Brachypodium distachyon*, an important model plant species, is also a member of the C3 grasses.

Plant breeding has successfully contributed to crop improvement and feeding the Earth’s increasing population since the beginning of the crop domestication process. Application of pre-genomics breeding technologies, combined with conventional breeding, have contributed significant yield improvement in various crops in the last century (Pérez-de-Castro et al., 2012). Further increasing crop yields to fulfill the increasing demand of a Malthusian-predicted population will be a daunting task due to low genetic gains and various abiotic and biotic stresses that threaten food security and impose a tremendous challenge for the next century of agriculture (Kujur et al., 2013). Despite their importance as animal feed and the basis of healthy meat, milk, and other products for human consumption, perennial C3 cool-season grasses have received limited genetic improvement efforts. Though research attention is highly varied, the next level of genetic improvement for all cool-season C3 grasses is necessary based on increasing demand and improvement status.

Conventional breeding methods still are the most applied and efficient methodologies, but are not enough to cope with the increasing demand for both food and feed. Genomics breeding, a recent invention adopted by breeders, promises a paradigm shift by enhancing the association study of genotype versus phenotype (Tester and Langridge, 2010). The mainstay of genomic breeding is high-throughput DNA sequencing, which, combined with other molecular technologies [i.e., high-throughput genotyping, constructing high density genetic maps for marker phenotype association, marker-assisted selection (MAS), breeding by design and genomic selection (GS)], will be able to bring a breakthrough in food and feed production (Peleman and van der Voort, 2003; Varshney and Tuberosa, 2007; McMullen et al., 2009; Lorenz et al., 2011). Genomic technologies and platforms are currently available in several C3 cereal grass species (Xu et al., 2005; Tuberosa and Salvi, 2006; Araus et al., 2008; Mochida and Shinozaki, 2010; Bohra et al., 2014); thus, genomics-assisted breeding is becoming popular in those species. However, resources for most of the C3 perennial grasses are yet to be developed. This is the time to equip breeding programs with advanced genomic tools to enhance genetic gains using desirable alleles from various gene pools of diversified genetic bases.

**FIGURE 1:**
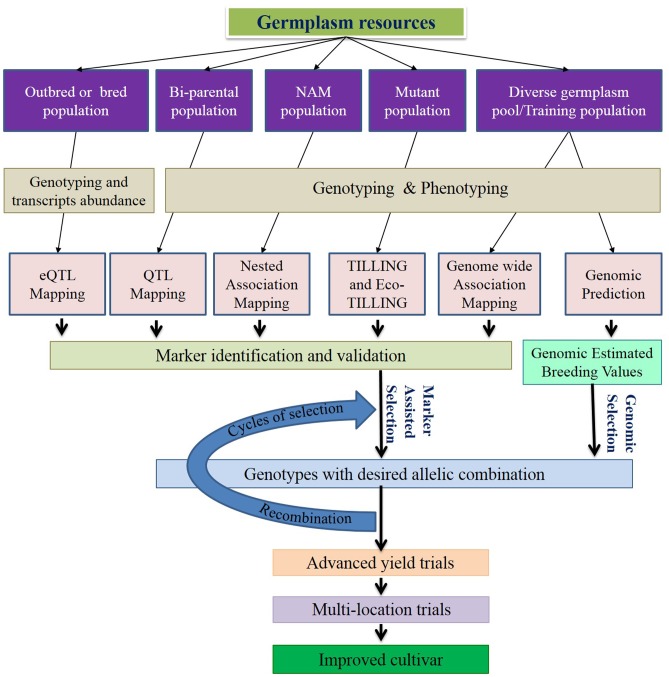
Schematic diagram of genomics-assisted breeding. Genomics technologies help enhancing marker trait association for marker-assisted selection (MAS) and genomic selection (GS). Both MAS and GS speedup selection cycles, increases precision and improves genetic gain per year. Selection and recombination will be repeated multiple times before the yield trials to increase the favorable allele frequency. Incorporation of genomics to the recurrent selection strategies substantiates the effectiveness of breeding program.

In this review, we discuss the recent and relevant advancements of genomics tools and resources, and their applications in C3 grass breeding (**Figure 1**), along with summarized information of genome and breeding behavior (**Table 1**), particularly cool-season perennials. The objective is to provide an updated status of various genomics technologies along with their potential use in cool-season perennial grass breeding programs.

**Table 1:** Name, genome information, and breeding behavior of commonly used cool-season C3 perennial grasses.

Common name	Scientific name	Genome size	Ploidy level	Breeding system	Chromosome number
Bromegrasses/Smooth brome	*Bromus inermis*	6.0–26.0 Gb	Diploid to decaploid	Open pollination	2*n* = (2*x* - 10*x*) = (14 - 70)
Kentucky bluegrass	*Poa pratensis*	6.9–12.80 Gb	Pentaploid to quindecaploid	Open pollination	2*n* = (5*x* - 15*x*) = (35 - 105)
Tall fescue	*Festuca arundinacea*	5.25–5.83 Gb	Hexaploid	Open pollination	2*n* = 6*x* = 42
Orchardgrass	*Dactylis glomerata*	4.31 Gb	Tetraploid	Open pollination	2*n* = 4*x* = 28
Perennial ryegrass	*Lolium perenne*	2.7 Gb	Diploid	Open pollination	2*n* = 2*x* = 14
Reed canarygrass	*Phalaris arundinacea*	4.96–5.17 Gb	Tetraploid/hexaploid	Open pollination	2*n* = (4*x* - 6*x*) = (28 - 42)
Timothy	*Phleum pratense*	NA	Diploid/hexaploid	Open pollination	2*n* = 6*x* = 42
Bentgrass	*Agrostis* spp.	2.7–2.8 Gb	Tetraploid	Open pollination	2*n* = 4*x* = 42
Intermediate wheatgrass	*Thinopyrum intermedium*	12.6 Gb	Hexaploid	Open pollination	2*n* = 6*x* = 42


*NA, the information about genome size was not found.*

## Genomics Tools and Resources

### Whole Genome Sequencing

The availability of the whole genome sequence of a crop immensely helps to unlock the genome for necessary improvement through breeding. A complete reference genome sequence can potentially be used for discovering genes and regulatory elements, as well as sequence variation in the targeted DNA region. With the enormous advancement of next-generation sequencing (NGS) technology, the whole genome sequence of several C3 grass species (*Brachypodium*, rice, millet, wheat, and barley) are available (Goff et al., 2002; Yu et al., 2002; Vogel et al., 2010; Bennetzen et al., 2012; Consortium, 2012; Jia et al., 2013; Ling et al., 2013; Marcussen et al., 2014). Due to cost reduction and technology advancement, other cool-season perennial C3 grass genome sequences will be within reach (Byrne et al., 2011).

The arrival of NGS technologies has dramatically changed genomics research. The new technologies have reduced sequencing cost more than a 1000 times (Muir et al., 2016). Thus, many laboratories have started developing their own sequencing facilities. New third-generation sequencing platforms enhance the sequencing process by increasing the read length up to 10,000 bp to substantially improve the assembly. They are available in PacBio RS (Pacific Biosciences^1^), Helicos (Helicos^2^) or Ion Torrent (Life Technologies^3^). Recently, a long-read sequencing technology called Moleculo technology was launched by Illumina. Moleculo technology yields more than 10 kb^4^ individual sequence reads. The pitfall of the long-read sequencing platform is the positive correlation of sequence size with error rate (Nagarajan and Pop, 2013).

The large and repetitive nature of most cool-season grass genomes is the fundamental drawback for constructing a long continuous assembly. Moreover, polyploidy, heterozygosity, and subgenome existence cause additional problems during assembly. Therefore, third-generation long sequencing reads are extremely valuable for developing high quality assembly in such genomes. A large-scale sequence structure of DNA can be determined by third-generation mapping technologies. Mapping technologies like the Irys system from BioNano Genomics, Hi-C mapping of Dovetail Genomics and Chromium instrument from 10X Genomics will be very useful to generate high quality long contigs. The combined use of physical mapping and whole genome shotgun sequencing of single flow-sorted chromosomes like wheat might be fruitful to assemble a high quality draft of complex genomes (Kopecký and Studer, 2014). Hybrid assembly strategies of short, high-fidelity reads together with long reads from different sequencing platforms using hybrid error correction or self-correction algorithms might yield 99.9% base call accuracy (Lee et al., 2016; Zimin, 2016).

Among the cool-season grasses, only the perennial ryegrass draft genome sequence is available until now (Byrne et al., 2015). Draft genome sequencing and development of a genomic toolbox are underway in intermediate wheatgrass (Dorn, 2017a,b). Genomic research and information on other grasses like tall fescue, Kentucky bluegrass, orchardgrass, and bentgrass are rapidly developing. Guided *de novo* assembly of the whole genome using other sequenced C3 grasses might be helpful to functionally characterize those genomes. The advancement of sequencing throughput at lower cost will tremendously enhance the whole genome sequencing of individuals in the population to explore genetic variations among them. This will help detect millions of SNPs and genome-wide variations. Such high density markers will enable identification of candidate genes or markers useful for enhancing selection efficiency and breeding gains in breeding programs.

### Transcriptome Sequencing

DNA sequencing and assembly of very large, complex polyploid grass genomes is still difficult with the currently available NGS technology. An alternative method for capturing the meaningful information of such genomes is whole genome next-generation transcriptome sequencing or RNA sequencing (RNA-seq) (Grabherr et al., 2011; Hirsch et al., 2014). Sampling from various developmental stages and/or tissues of diverse crops’ genotypes enhances the generation of global transcript sequences along with variably expressed candidate genes (NCBI, GEO database). This has proven to be a powerful technology (Wang et al., 2009; Pingault et al., 2015) to capture differential gene expression, determine exon/intron boundaries (Lu et al., 2010), study alternative splicing (Xie et al., 2015), identify post-transcriptional changes (de Alba et al., 2015), study transcription factors (Xu et al., 2014), microRNA, ribosomal RNA, transfer RNA, and small nuclear RNA (Rinn and Chang, 2012). This method has been successfully used to identify simple sequence repeats (SSRs) (Chapman, 2015) and SNP markers for assisting breeding in tall fescue (Talukder et al., 2015). The identification and usefulness of microsatellite markers using next-generation sequencing data was found promising in perennial ryegrass (Honig et al., 2017). In other cool-season perennial grasses, transcriptome sequences or other next-generation sequences might also be useful for genetic analysis and marker development. In a comparative transcriptome analysis, a stress-responsive protein has been identified in Lolium/Festuca species (Czaban et al., 2015). Recently, transcriptome analyses were done to study dwarfism to enhance dwarfing breeding in Kentucky bluegrass (Gan et al., 2016), drought stress response in creeping bentgrass (Ma et al., 2017), water stress (Talukder et al., 2015), lead (pb) stress (Li et al., 2017), and endophyte response in tall fescue (Dinkins et al., 2017), and to develop a unigene reference as a molecular breeding resource in *Phalaris* (Baillie et al., 2017). Markers developed from the reference transcript corresponding to the protein might be useful for MAS for stress tolerance. Approximately 72–87% of grass genes are predicted to be collinear (Mayer et al., 2009). Transcriptome sequences can be used to compare the conserved synteny of sequenced C3 grasses for gene/transcriptome quantification (Kopecký and Studer, 2014). Similarly, using the reference transcripts of identified SSRs or SNPs, functional importance as well as localized gene function might be explored for genomics-assisted breeding in understudied C3 perennial grasses (Talukder et al., 2015) along with best studied ones.

An emerging new approach called genome zipping has been developed for under studied and complex grass genomes. This approach works based on the high degree of synteny among the Poaceae grasses along with numerous genomics resources of various grass species. It usually identifies and organizes the syntenic region of sequenced genomes based on a genetic linkage map of the targeted species. The information from sequenced genomes is then integrated into an ordered gene model and resolves species-specific local arrangement (Pfeifer et al., 2013; Poursarebani et al., 2013; Kopecký and Studer, 2014). Among the cool-season grasses, the genome zipper approach was first implemented in perennial ryegrass and was proven useful for map-based cloning and QTL fine mapping (Brazauskas et al., 2013; Pfeifer et al., 2013; Arojju et al., 2016). Using assembled reference transcripts of NGS-based transcriptome sequences, the genome zipper approach would be very useful to generate a genome draft. The retrieved functional gene and marker information will enhance genomics-assisted breeding.

### Mutational Genomics

Genetic variation is the pillar of plant breeding success. Various techniques have been developed and used to create mutant collections. The transferred DNA-tagged lines and transposon-tagged lines have been used to develop mutant collections in *Arabidopsis* (The *Arabidopsis* Information Resource^5^) and rice (International Rice Functional Genomics Consortium). RNA interference was used to create gene-specific mutant collections in *Arabidopsis*^6^. Using a reverse genetics approach called Targeting Induced Local Lesions in Genomes (TILLING), the allelic variations of an artificial mutant collection were identified (Till et al., 2003). A similar approach to identifying allelic variation in a natural germplasm collection is called ecotype TILLING (EcoTILLING) (Comai et al., 2004). Both methods use CEL 1 or Endo 1 to recognize and cut double helix DNA to identify allelic variants in a certain genomic region (Till et al., 2004). The frequency and efficiency of these methods significantly rely on available NGS sequences yielded from gene expression studies (Pérez-de-Castro et al., 2012). Both methods were successfully implemented in C3 Grasses. TILLING has been implemented in barley (Caldwell et al., 2004), while EcoTILLING has been applied to rice (Kadaru et al., 2006), wheat (Wang et al., 2008), and barley (Mejlhede et al., 2006). In recent years, mutagenesis has advanced significantly. Various site-directed or site-specific nucleases (SDNs/SSNs), such as zinc-finger nucleases, TALENs or CRISPR/Cas9, have been developed and used as mutagenic agents to discover potential alleles using forward and reverse genetics approaches (Pradhan et al., 2015; Nogué et al., 2016). Most cool-season grasses have large complex genomes with scarce genomic information. Thus, the successful application of these techniques should be followed by QTL identification.

### High-Throughput Molecular Markers

Plant breeders predominantly like SSR and SNP markers for marker-assisted breeding. NGS technology has made it possible to rapidly sequence multiple individuals for minimum cost. Powerful computational pipelines and various software are greatly enhancing the mining of NGS sequences for genetic variations, i.e., SSRs and SNPs. Due to abundance, reproducibility, amenability to automation and tremendous cost effectiveness, SNPs are becoming the marker of choice to plant breeders (Pérez-de-Castro et al., 2012). Massive re-sequencing and genome-wide SNP discovery have been performed in *Arabidopsis* (Weigel and Mott, 2009). Aligning reads from the 3000 Rice Genome Project, 20 M SNPs have been identified and provided in a SNP-Seek system^7^ (Alexandrov et al., 2015). Recently, a 90k SNPs genotyping array was developed in wheat and found to be well distributed in the genome (Wang et al., 2014). More recently, 4 M SNPs have been identified and characterized in the wheat genome. These are available in a generic genome browser, GBrowse, at www.wheatgenome.info (Lai et al., 2015). SNP identification is challenging in complex polyploid and/or outcrossing heterozygous genomes due to mis-assembly and false positive SNP identification (Mason, 2015). The presence of reference genomes of the species facilitates the reduction of alignment difficulties and thus increases the accuracy of SNP identification along with genome position (Ruperao and Edwards, 2015). SNP identification efficiencies of crops with no prior genome information can be facilitated by incorporating several paired end sequencing techniques. Other ways to facilitate SNP identification are transcriptome sequencing, genotype by sequencing (GBS), and exon capture. Transcriptome sequencing has been applied to SNP and SSR identification in many crops with limited genetic information and no reference genome sequences, i.e., tall fescue (Talukder et al., 2015), red clover (Yates et al., 2014), camelina (Mudalkar et al., 2014), oaks (Torre et al., 2014), turmeric (Sheeja et al., 2015), mung bean (Chen et al., 2015), and lupin (Kamphuis et al., 2015). Identified markers, as well as their reference transcript, might be aligned to the closely related sequenced genome for further exploration of the targeted markers (Talukder et al., 2015).

Genotype by sequencing is a rapid, robust and cost-effective genotyping technology that performs genome-wide molecular marker discovery by reducing genomic complexity. Application of this technology in plant species without prior genetic resources for genomic research has paved the way for researchers to adopt GBS as a high-throughput marker technology for orphan crops as well. This method has been validated to be used in diversity (Elshire et al., 2011) and association studies (Hegarty et al., 2013; Morris et al., 2013) as well as advanced breeding application (Poland et al., 2012; Poland, 2015). A cost-effective targeted amplicons-based GBS approach was found very effective for genotyping perennial ryegrass and Italian ryegrass (Pembleton et al., 2016). Along with marker identification, GBS is a powerful tool to calculate genome-wide allele frequency of a particular locus. This can be a very effective tool for various forage, lawn, and turf grass breeding (Byrne et al., 2013).

Restriction site-associated DNA sequencing (RADSeq) is also a genotype-by-sequencing approach to marker discovery and can identify widely distributed markers across the genome using NGS technology (Baird et al., 2008; Hohenlohe et al., 2010). Akin to GBS, it also reduces the genome complexity by subsampling the restriction site of specific enzymes and can provide genomics-scale insights for orphan crops with no prior genomic information available. Many SNP-based genetic maps and association studies have been reported using a RAD sequencing platform in understudied grasses (Chutimanitsakun et al., 2011; Hegarty et al., 2013; Wang et al., 2013; Slavov et al., 2014; Varshney, 2015; Wang F. et al., 2015).

Exome capture is a newly emerging genomics tool for marker identification. It is rapid, cost effective and applicable to forage, lawn, and turf grasses with limited genetic/genomic information. Exome capture was initially reported in human genomics. In this technology, only protein coding regions of a genome are captured and separated by hybridizing genomic DNA with biotinylated oligonucleotide probes complementary to targeted exons, thus reducing the unnecessary junk part of the genome for sequencing (Choi et al., 2009; Ng et al., 2009). Exome capture and SNP identification has been successfully performed in many crops, i.e., barley (Mascher et al., 2013), rice (Henry et al., 2014), and wheat (Winfield et al., 2012; Allen et al., 2013). Capturing assays developed for one species can be applied to related species to enrich the genomic region (Mascher et al., 2013). Following this idea, switchgrass (*Panicum virgatum*) exome capture probes were used to perform exome capture sequencing in bermudagrass (*Cynodon dactylon*) genotypes for SNP calling and GWAS study (Malay Saha, Noble Research Institute, LLC). Both the abovementioned species belong to the C4 grasses; however, non-model C3 grass species can also be studied and compared with other C3 grasses with existing reference sequenced genomes.

### High Density Genetic Maps

The advancement of genomics technology has significantly enhanced the development of high density genetic maps even in understudied crops with large, complex genomes. Integration of NGS and high-throughput genotyping platforms significantly increased marker density in genetic maps. Construction of SNP-based genetic maps is faster and effective for crops with intense genetic information. The Illumina GoldenGate has been the most widely used platform for SNP genotyping, while Sequenom MassARRAY platform-based SNP-typing assays are becoming popular as well (Oliver et al., 2011; Chagné et al., 2015). SNP-based high density linkage and transcriptome map construction using NGS data has been successful in wheat (Wu et al., 2015; Holtz et al., 2016), rice (Xie et al., 2010; Zhang et al., 2015), maize (Liu et al., 2010; Mahuku et al., 2016), barley (Chutimanitsakun et al., 2011; Obsa et al., 2016), perennial ryegrass (Pfender et al., 2011; Paina et al., 2016; Velmurugan et al., 2016), orchardgrass (Zhao et al., 2016), intermediate wheatgrass (Kantarski et al., 2017), and zoysia grass (*Zoysia japonica*) (Wang F. et al., 2015). NGS-derived SNP-based genetic maps are useful for comparative mapping and have great potential for cool-season perennial grasses with limited genomic information available. These SNP markers can be aligned to genomes or linkage maps of closely related C3 grasses like *Brachypodium*, rice, wheat, and barley. The comparison of high density SNP maps was useful for barley with wheat and rice (Close et al., 2009; Sato et al., 2009), switchgrass with foxtail millet (*Setaria italica*) (Daverdin et al., 2014) and muscadine grape (*Vitis rotundifolia*) with European bunch grape (*Vitis vinifera*) (Owens, 2015). Comparative genetics analysis can also be performed using SSRs and other molecular-marker-based maps (Paolucci et al., 2010; Hudson et al., 2012; Dierking et al., 2015). Recently, genome-wide SSR sequences were detected from nine completely sequenced grass species (*Oryza sativa* L. ssp. japonica, *Oryza sativa* L. ssp. Indica, *Zea mays*, *Sorghum bicolor*, *Brachypodium distachyon*, *Setaria italica*, *Phyllostachys heterocycla*, *Triticum urartu*, and *Aegilops tauschii*). The descriptions of the SSRs were provided in the Poaceae SSR Database^8^ in terms of abundance, density, base ratio of different motifs, and genomic elements, i.e., exon, intron, and UTR (Wang Y. et al., 2015). Genetic maps combining both SSRs and NGS-SNPs would thus be very effective for MAS. Co-localized SSRs and SNPs will provide additional information and validation through comparative genomics and genetics. Genetic maps using mixed marker systems have been reported and found promising in wheat (Talukder et al., 2014; Wu et al., 2015), pear (*Pyrus* spp.) (Wu et al., 2014), common bean (*Phaseolus vulgaris* L.) (Schmutz et al., 2014), faba bean (*Vicia faba* L.) (Satovic et al., 2013), orchardgrass (Zhao et al., 2016), and many other crops. SNP identification in outcrossing polyploids is very critical. Identification of a high number of false positive SNPs is highly likely. False positive SNPs can be an obstacle during genetic linkage map construction. SSR markers thus can be used as references to construct accurate linkage maps. However, construction of high density linkage maps using only SSR markers will be expensive and time consuming. Thus, high density genetic linkage maps using a mixed marker system might be very useful for cool-season perennial grasses like tall fescue, orchardgrass, Kentucky bluegrass, perennial ryegrass and bentgrass.

### Phenomics for Genomics-Assisted Breeding

Breeding success relies on capturing the best genetic variation from the germplasm resources. Breeders need to manage very large breeding populations to develop superior varieties. NGS-based high-throughput genotyping has facilitated a way to incorporate 1000s of genotypes in the mapping population. The advancement of high-throughput phenotyping (HTP) technology cannot keep pace with the high-throughput genotyping and thus has been considered a limiting factor for next-generation genomics-assisted breeding (Houle et al., 2010). Recently the importance of HTP has been emphasized. There are fully automated and precisely controlled phenotyping platforms available in some public plant research institutions, i.e., the USDA^9^^,^^10^, the Australian Plant Phenomics Facility^11^, and the European Plant Phenotyping Network^12^, with remote sensing facilities to monitor plant growth and performance (Araus and Cairns, 2014). Variability of environment, soil and drought conditions in the field cannot be provided to plants growing in pots under controlled-environmental conditions. Phenotyping for drought in a pot is incredibly difficult. The gradual decline of soil moisture in the field is associated with increased mechanical encumbrance, which is difficult to imitate in pots (Cairns et al., 2011). Therefore, the translational ability of controlled-environment-phenotyping results in the field is very low. Moreover, the variety development process involves multi-environment trials where plants are exposed to various stresses throughout their life cycle. As a result, an increased effort has been made to implement HTP in the field. Usually, HTP platforms rely on image capturing to phenotype plants. Several review articles have been published describing imaging techniques, novel sensors, image analysis, modeling, robotics, and data mining for both ground-based and aerial systems HTP platforms (Cobb et al., 2013; Costa et al., 2013; Araus and Cairns, 2014; Goggin et al., 2015; Humplík et al., 2015). Various phenomobiles have been used in recent years as ground-based HTP. Phenomobiles are modified vehicles with global positioning systems (GPS) combined with various sensors. This type of phenomobile is effective for small-scale breeding programs to produce plot-level data. The phenomobile used in cotton field phenotyping contains four sensors for measuring canopy height, reflectance and temperature on four rows simultaneously at a rate of 0.84 ha per hour (Andrade-Sanchez et al., 2014). A mobile platform with hyperspectral passive spectrometer to predict crude protein in wheat, tall fescue, and bermudagrass was also successful (Pittman et al., 2016). Prediction of biomass yield, the most important trait for C3 grass breeding will be more accurate. The limitations of phenomobiles are time consumption and data processing. Compared to ground-based HTP, aerial HTP is much faster and can characterize all plots in a trial within a minute using various platforms, i.e., phenotowers (Rascher et al., 2011), blimps (Losos et al., 2013), and unnamed aerial platforms (UAP) such as airplanes and polycopters. However, aerial HTP involves high cost, and superior mechanical and data processing skills. Overall, HTP clearly demonstrates the potential of non-destructive phenotyping with precision and pace in a tractable way (Poland, 2015). Upon application in breeding programs, HTP will balance the genotype-to-phenotype dataset and provide raw materials for next-generation breeding.

## Genomics Approaches For C3 Perennial Grass Breeding

### Genome-Wide Association Studies (GWAS)

Plant breeders struggle to capture and transfer genetic variability of target traits. In most cases, the existing genetic variation remains untapped due to lack of resources in breeding programs to allow the utilization of variability in the available germplasm. High-throughput genotyping platforms permit genome-wide marker detection for high resolution marker profiling and are already being used in a few C3 grasses, i.e., orchardgrass (Zeng et al., 2017) and perennial ryegrass (Ruttink et al., 2015), for genome-wide association studies (GWAS). GWAS is an excellent tool that has been used to identify genetic variants associated with traits of interest. GWAS provides understanding of genome function as well as allelic architectures of complex traits (Huang et al., 2010). With advanced genotyping technology, GWAS is becoming a very powerful tool for crop breeding enhancement. In the case of autogamous homozygous or clonally maintained allogamous species, a GWAS panel might be a permanent resource for the crop (Huang and Han, 2014). Nested Association Mapping (NAM), another genome-wide association strategy that simultaneously exploits the advantage of linkage analysis and association mapping, can be an excellent tool to capture marker trait association (Yu et al., 2008). There is no report of NAM in any C3 cool-season perennial grasses. The major limitation is that it is generally not possible to produce inbred lines in outbreeding crops and maintain seeds for the research community. Pseudo F2 families’ development and clone maintenance of those families can be done in these crops. A modified chain-cross protocol was used to develop a NAM population in switchgrass, a C4 outcrossing polyploid species (Ali et al., 2016). Developing and maintaining such population are usually expensive and time consuming. However, once developed, the population could be an asset for grass breeding programs. The cool-season C3 perennial grasses are mostly allogamous; therefore, capturing linkage disequilibrium (LD) of those crops requires high resolution marker information in the association panel. Simultaneously, the size of panel should be large enough to maximize the statistical power for rare allele detection. Genome-wide association studies have been implemented in perennial ryegrass and tall fescue among the allogamous C3 grasses (Skøt et al., 2005; Yu et al., 2013, 2015; Aleliūnas et al., 2015; Lou et al., 2015). Using high-throughput genotyping platforms, i.e., GBS and RAD seq, an enormous amount of SNPs can be identified for GWAS to capture the LD decay in C3 grass panels with limited genetic information available. Thus, GWAS can be a powerful tool for those orphan crops for practical genomics-assisted breeding.

### Mapping QTL and Identification of Markers Linked to the Traits

Next-generation sequencing significantly enhanced genotyping platforms for high resolution marker identification and accommodation in the genetic map for QTL study (**Figure 1**). It is possible to detect markers tightly linked to the target traits by increasing the genome coverage of marker density in optimum size mapping population. The effective size of the mapping population is crucial in capturing recombinant events. Increasing markers in a small population will make many of the markers redundant or uninformative. Along with the increasing accuracy of QTL detection, the number of QTL studies is growing at an impressive pace. During the last 22 years, 133 QTL studies on yield and 361 QTL studies on disease resistance reported 1,600 and 4,300 QTL, respectively, in wheat (Salvi and Tuberosa, 2015). It has been clear from empirical analysis that a large number of QTL have a smaller effect while a smaller number have a larger effect (Flint and Mackay, 2009; Kliebenstein, 2010). This observation complies with the Fisher-Orr model, which describes strong alleles segregating for a loci quickly get fixed and disappear from the population while weak alleles keep segregating (Orr, 2005). Thus, complex traits like yield in elite cultivars are prone to be detected with minor effect QTL. However, many major effect QTL also have been identified, validated, cloned and successfully utilized by breeders for yield, plant height, heading date, flowering time, drought tolerance, disease resistance, salt tolerance, and nematode resistance in many crops, i.e., rice, wheat, sorghum, chickpea, maize, and soybean (Xing and Zhang, 2010; Griffiths et al., 2012; Salvi and Tuberosa, 2015). Besides the elite members of the C3 grasses, QTL studies also have been reported in perennial ryegrass (Armstead et al., 2004; Jensen et al., 2005; Muylle et al., 2005; Turner et al., 2006; Sim et al., 2007; Schejbel et al., 2008; Pearson et al., 2011; Khaembah et al., 2013; Ma et al., 2014; Paget et al., 2014; Kundu et al., 2015), meadow fescues (Ergon et al., 2006; Alm et al., 2011), creeping bentgrass (Jespersen et al., 2016), and orchardgrass (Zhao et al., 2016). Few linkage maps have been reported in tall fescue (Saha et al., 2005; Dierking et al., 2015). Recently a consensus genetic map using GBS markers has been published in intermediate wheatgrass (Kantarski et al., 2017). However, there is not much QTL mapping information available for other cool-season perennials.

Overall, QTL information in various traits has tremendous potential to enhance plant breeding. Using NGS-based SNP information in accordance with SSRs may identify high powered QTL and resolve the linkage phase between marker and the QTL alleles.

A new strategy of QTL mapping based on abundance of measurable transcript levels in diverse mapping populations is emerging. In this method, DNA polymorphisms can be identified that control single/multiple gene expression levels (Schadt et al., 2003). Advancement of sequencing technologies, accompanied with reduced cost, opened the door for transcriptome sequencing or expression microarrays of large biparental/GWAS populations. Put simply, expression levels of transcripts/genes are measured and used as the phenotype to find the marker-transcript association. There is no report of eQTL study in C3 perennial grass. With next-generation sequencing help, eQTL holds great promise for effective marker identification to enhance MAS (**Figure 1**).

### Marker-Assisted Selection

Marker-assisted selection can enhance a breeding program by selecting plants in an early generation, thereby reducing breeding time (**Figure 1**). It is the oldest and most widely used technology to be considered genomics-assisted breeding. The success of MAS relies on identification of markers tightly linked with the genes or genomic region (QTL) of a target trait. With the recent advancement of the NGS-based genotyping platform, it is possible to identify markers very close to the target region. The rule of thumb for MAS is “The closer the marker to the gene/QTL, the more effective the MAS.” The linked markers are applied to select introgressed individuals with desired allelic combinations in early generations when phenotypic evaluation is not fruitful. Recombination may displace a closely linked intergenic marker; thus intragenic markers called functional markers are always preferable for MAS. In this case it is highly unlikely to displace the marker from the gene through recombination. Because of abundance, randomness, and polymorphism in the genome, NGS-based SNPs have a high chance of being linked as functional markers. Similarly, NGS markers can identify individuals with critical recombination break points to eliminate linkage drags for important traits (Fukuoka et al., 2009; Venuprasad et al., 2012). Therefore these markers enhance gene-assisted breeding, eliminating the possibility of losing the trait by recombination as well as expelling the linkage drag from desired elite lines (Pérez-de-Castro et al., 2012; Varshney et al., 2014).

Traits with both simple and complex inheritance (i.e., disease, pest and insect resistance; heat, drought and salt tolerance; and grain and biomass yield and quality) are being used for MAS in various C3 grasses. Breeders usually use MAS for introgression of minor alleles to the elite germplasm, gene pyramiding and improve complex traits with phenotyping difficulties (Varshney et al., 2014).

C3 grasses with limited genetic resources can be easily studied with NGS-based markers and background genetic information retrieved through collinear study with other well-studied C3 grasses. A well-studied marker system has already been developed in perennial ryegrass suitable for MAS (KöLliker et al., 2005; Rajicic et al., 2015). Other cool-season perennial grasses can be syntenized with *Brachypodium*, rice, and wheat for validation and functional information of the linked marker. A few 1000 SSRs and SNPs have been identified in tall fescue by Talukder et al. (2015). They found very good distribution in the *Brachypodium* genome. They also found more than 60 percent genome similarity between the tall fescue transcriptomes and the *Brachypodium* genome. The Saha group (Grass Genomics Laboratory) at the Noble Research Institute is in the process of building a marker database for tall fescue, which might also be useful for other cool-season C3 perennial grasses. SSR markers associated with forage digestibility were identified and used in a MAS program. Synthetic populations have been developed by random mating of MAS genotypes from a breeding population of tall fescue. The population looks promising. Evaluations of the synthetic population at multi-location field trials are in progress. Chloroplast markers discriminating Mediterranean and Continental morphotypes were identified in tall fescue and are being used at the Noble Research Institute for tall fescue morphotype identification. Most cool-season perennial grasses are outcrossing in nature. Allele accumulation by recurrent selection is time and labor intensive for outcrossing species. Tightly linked markers would help reduce the number of genotypes in every cycle and enhance phenotyping efficiency, as well as intensify favorable alleles in the population. Thus there is great promise for MAS breeding in the cool-season C3 grasses. Many of the perennial C3 members are not genetically well documented. Meanwhile, the success of genomics-assisted breeding solely depends on the gene or QTL identification for various traits of interest. Frequent genetic study of marker/gene/QTL identification in those orphan grasses will definitely enhance breeding programs for necessary improvement.

### Genomic Selection (GS)

Genomic selection is a form of MAS that attempts to eliminate the limitations of MAS. Usually MAS success depends on QTL detection, which has been questioned for certain limitations in both biparental and genome-wide association mapping strategies. Firstly, the allelic distribution of a breeding program cannot be covered by a biparental mapping population, and generated information is merely applicable for the breeding program without proper validation. Moreover, the accuracy and marker effect are mostly overestimated in biparental QTL mapping. Genome-wide association studies reduced the limitation of population development and allele frequency limitation in biparental QTL mapping; however, low heritability, few large-effect QTL, confounding population structure and QTL with overestimated effects are still limitations of MAS (Holland, 2007; Heffner et al., 2009).

In GS, all marker data in the population are utilized as breeding value predictors. A prediction model is developed based on marker information, phenotyping information and pedigree information of the training population, which provides genomic estimated breeding values (GEBVs) for all genotyped germplasm in the breeding population to predict plant performance in the breeding program (**Figure 1**) (Meuwissen et al., 2001). Prediction accuracy relies on the genetic relationship between the training population and the breeding population, phenotyping accuracy, age of the training population, heritability of the predicted trait and breeding population structure (Jannink et al., 2010; Crossa et al., 2014; Varshney et al., 2014). Providing pedigree information and genome-wide high density marker deployment can definitely increase the prediction accuracy of GS. As we discussed earlier, many C3 grass species lack substantial genetic information. However, GS does not require prior genetic information, and, thus, the technique is directly applicable for those grass breeding programs with limited or no genetic information. The main expected benefits of GS for these crops are to increase selection efficiency, increase average genetic gain per year, reduce time of breeding cycle and reduce breeding cost. Almost all the commonly used C3 perennial grasses are outcrossing, and limited knowledge of LD and effective population size (*N_e_*), constraints in SNP discovery and validation, prediction accuracy, and increased annual inbreeding rate are the major limitations in GS of the target grasses (Forster et al., 2014). Effective GBS method development for determining allelic dosages is still an issue (Hayes et al., 2013). Another problem of such crops might be the stagnation of the training population. Considering the economic investment, most programs may use part of their breeding program as the training population which might have experienced directional selection for a few generations; thus genetic variance of the markers might be reduced (Simeão Resende et al., 2014). The complexity of controlling genetic variation might be navigated by using accurate genotyping assay and a higher number of markers (Fè et al., 2016; Grinberg et al., 2016). In this regard, recently developed SNP identification pipelines that are effective for SNP identification in polyploid genomes might be helpful. Being outcrossing, most of the cool-season perennial grasses might have lower LD and greater complexities to study genetic variations. The problem of lower LD in perennial ryegrass could be reduced by using structure and family information in the breeding population (Fè et al., 2015). Shortening breeding cycle time in GS may enhance the annual inbreeding rate (Lin et al., 2016). To control this type of inbreeding in GS of outbreeds, Lin et al. (2017) proposed three types of heuristic approaches, i.e., controls during mate allocation, during selection, and simultaneous selection and mate allocation. The lower prediction accuracy of GS might be increased by increasing and updating the population at frequent intervals. However, larger populations with continuous updates will increase the cost per unit gain. Estimating breeding value of an individual based on performance of its relatives may provide a reasonably higher genetic gain per year, as well as prediction accuracy. This approach may be problematic in cool-season perennial grass breeding due to unknown pedigree of many individuals. Moreover, performance of the individual family needs to be measured as plot-family mean basis thus; higher genotyping and phenotyping cost will be involved in this system. Prediction accuracy and genetic gain can be increased to a reasonable level in the miniplot genotyping system by increasing plot number and reducing selection intensity. However, that may increase the cost of phenotyping as well (Lin et al., 2017). In reality, it is highly likely that the genotyping cost will be reduced at a significantly higher pace than the phenotyping cost. The breeding programs will be more organized in future in keeping track of pedigree. Therefore, single genotype genotyping might yield more economic gain per unit of genetic gain. However, prediction accuracy comparison of plot-family-based allele frequency of miniplots using family-trait mean and individual allele of single genotype genotyping using individual trait were found equivalent in a GS model (Munoz, 2017). A relatively higher number of alleles are accommodated in the prediction model for family-based allele frequency. This type of prediction model might be highly useful because developing a synthetic variety based on a greater amount of allelic information might increase the sustainability of a released cultivar. Till now, there have not been many reports about the application of GS in C3 cool-season perennial grasses except perennial ryegrass, but with the advent of NGS-based genotyping platforms, breeders will be encouraged to adopt GS for varietal improvement.

## Conclusion

The advancement of genomics resources and tools has shown tremendous enhancement in the breeding program. Combined with conventional breeding, genomics tools and resources will dramatically hasten the achievement of expected genetic improvement of important grasses, most significantly some genetically orphan cool-season C3 perennial grasses. In the near future, breeding programs will be large enough to maintain 1000s of lines with rich genomic resources. Concomitantly, focus has already been given to HTP (Phenomics). Once genomics and phenomics are on the same page and with fully developed data management systems, vast knowledge about genomes will be fully mined; thus, genomics tools will uphold the promise of ensuring food security for the population.

## Author Contributions

ST has written the manuscript. MS has contributed to provide idea, information, and organization for writing, as well as writing some portion of the manuscript.

## Conflict of Interest Statement

The authors declare that the research was conducted in the absence of any commercial or financial relationships that could be construed as a potential conflict of interest.

## Footnotes

1 http://www.pacificbiosciences.com

2 http://www.helicosbio.com

3 https://www.thermofisher.com/us/en/home/brands/ion-torrent.html

4 https://www.illumina.com/science/technology/next-generation-sequencing/long-read-sequencing.html

5 http://www.arabidopsis.org

6 http://www.agrikola.org

7 http://snp-seek.irri.org/

8 http://biodb.sdau.edu.cn/pssrd/index.html

9 https://nifa.usda.gov/sites/default/files/resources/Whitepaper_finalUSDA.PDF

10 http://www.wheatgenetics.org/downloads/Projects/HTP_ProjectNarrative_20130219.pdf

11 http://www.plantphenomics.org.au/

12 http://www.plant-phenotyping-network.eu/eppn/structure
